# Biochemical curiosities: Piquing students’ interest in biochemistry by exploring evolution’s pursuit of the improbable

**DOI:** 10.1038/s44319-024-00259-7

**Published:** 2024-09-13

**Authors:** Sunil Laxman

**Affiliations:** Institute for Stem Cell Science and Regenerative Medicine (inStem), GKVK Post Bellary Road, Bangalore, Karnataka 560065 India

**Keywords:** Careers, Metabolism, Methods & Resources

## Abstract

A teaching course based on seemingly improbable curiosities could attract students’ interest in the magic of biochemical pathways and their evolution.

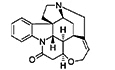

“Curiouser and curiouser!” cried Alice (she was so much surprised, that for the moment she quite forgot how to speak good English).—Lewis Carroll, *Alice in Wonderland*

Curiosity—(1) a desire to know or learn, (2) an unusual and interesting thing or person.

Life is a myriad of well-orchestrated chemical processes that occur in cells, and the study of these chemical conversions within and of living organisms is called bio(logical) chemistry. However, an unfortunate feature of biochemistry and its contemporary teaching in schools and universities is its emphasis on the minutiae of molecular mechanisms, at the cost of ignoring life itself. Most undergraduates are struck with paralyzing terror when they encounter biochemistry and the sheer drudgery of memorizing the details of metabolic or synthetic pathways that studying biochemistry entails. Hordes of life science students go through college without learning to appreciate the enormous spectrum of chemistry that living cells continuously perform, and never stop to wonder why such reactions exist in the first place.

“Most undergraduates are struck with paralyzing terror when they encounter biochemistry and the sheer drudgery of memorizing the details of metabolic or synthetic pathways that studying biochemistry entails.”

Indeed, biochemistry comprises of many improbable reactions and synthetic pathways that nonetheless follow an impeccable functional logic shaped and optimized by evolution. Many molecules and reactions have been preserved in bacteria, archaea, fungi, plants, and animals throughout hundreds of millions of years. On the other hand, some molecules are so unique that only a few select organisms synthesize them through reactions so improbable that they would be impossible to imagine were they not true. Arthur C. Clarke could have thought of biochemistry when he formulated his famous third law: “Any sufficiently advanced technology is indistinguishable from magic.”

“Arthur C. Clarke could have thought of biochemistry when he formulated his famous third law: “Any sufficiently advanced technology is indistinguishable from magic.”

## A new approach to teaching biochemistry

**Figure 1:**
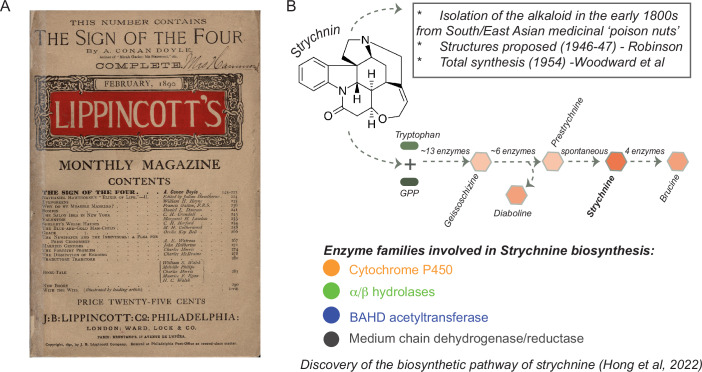
Strychnine: a poison with improbable biosynthesis. (**A**) The original publication of the second Sherlock Holmes novel, *“The sign of four”*, where strychnine’s sinister reputation as a deadly poison becomes legendary. Toronto Public library under a CC-BY-SA 2.0 license. (**B**) The chemical structure of strychnine - *‘the most complex molecule known for its size’* - along with a summary of its long history and a simplified schema of the biosynthetic pathway discovered in 2022 (Hong et al, 2022).

During many discussions, my colleague Padmanabhan Balaram and I concurred that nothing in evolution would make sense without biochemistry, nature’s unending, messy pursuit of chemical perfection. We further reasoned that to make students appreciate biochemistry, we might adopt an educational approach based on teaching curiosities: biochemical mechanisms that seem to be improbable. Take, for example, strychnine, the poison of choice for many a murder in literary and celluloid fiction (Fig. 1). It is an alkaloid naturally produced only by a small clade of sub-tropical trees of the genus *Strychnus*, and it is one of the most complex molecules known (Seeman and House, 2022). The pathway to synthesize this molecule is extremely elaborate and it was only recently fully described (Hong et al, 2022), 200 years after strychnine was first isolated. It is so improbable that it raises the question of why such a complex pathway might have evolved in the first place and for what purpose.

“… to make students appreciate biochemistry, we might adopt an educational approach based on teaching curiosities: biochemical mechanisms that seem to be improbable.”

This extravagant series of enzymatic reactions (Hong et al, 2022) allows students to study the hydrolysis, decarboxylation, oxidation, and reduction reactions that are required to transform a simple precursor to the complex final product. Next, one could move the focus from the individual biochemical reactions to the enzymes—and the genes that encode these enzymes—and ask how such a series of transformations could evolve over time by co-opting enzymes from other pathways or other functions. This raises the question of if and how it might be possible to engineer this metabolic pathway into a bacterial or yeast cell to produce this molecule.

Another curiosity could be something ubiquitous and well-known, such as the reckless, almost improbable electron transport chain that puts cells at grave risk of oxidative damage, even as it allows plants to harness the energy of the sun, and cells to generate ATP. Such examples nucleated our idea that “improbable biochemical reactions” or ‘biochemical curiosities’, could become the basis for a university course in advanced biological chemistry and chemical biology. This course has now been taught for four years by Padmanabhan Balaram and myself. Using three modules from the course as examples, I illustrate how ‘biochemical curiosities’ can be used to teach biochemical principles and their relevance, and help students to appreciate the chemistry of life.

## Building courses through curiosities

Mid-way through the first iteration of the course in early 2020, Covid-19 spread around the globe. Institutes shut down, and classes eventually resumed online. Since the pandemic was on everyone’s mind, the virus and the breathtaking pace of understanding it became the biochemical curiosity for the course. We began the course with the mechanism of viral entry into the target cell that requires the viral-spike protein to bind the ACE2 receptor. This allowed us to discuss mechanisms of protein–protein interactions and how this results in the exposure of a proteolytic cleavage site—the (in)famous ‘mutated’ furin cleavage site—on the SARS spike protein that gives this virus an incredibly efficient rate of infection (Coutard et al, 2020).

Students would then learn about protease function and explore the biochemical origins of proteases, and whether protein breakdown or synthesis might have evolved first. In exercises, they would quantify the relative effectiveness of natural proteolysis versus accelerated proteolysis by proteases, and understand how ubiquitous the hydrolysis mechanisms used by proteases are. For more entertainment, students could speculate on the evolution of biochemical mechanisms based on the murky possibilities of laboratory versus sea-food market origins of the virus (Balaram, 2021).

One recurring module in the course is built around cholesterol, a molecule everyone is familiar with—in the sense that familiarity breeds contempt—and still knows little about, other than incorrectly assuming it should be avoided in the diet. Some may vaguely recollect that cholesterol is related to steroid and sex hormones, which control many aspects of physiology, development, and reproduction—it is, in fact, the precursor of all steroid hormones. But the curiosity of cholesterol increases once students learn that it is one of the most important components of all eukaryotic membranes, and begin to ponder how and why. Functionally, cholesterol helps to organize lipid domains within membranes, which can be used to illustrate the many forms of biological organization and diversity that membranes enable.

“One recurring module in the course is built around cholesterol, a molecule everyone is familiar with […] and still knows little about, other than incorrectly assuming it should be avoided in the diet.”

More evocatively, cholesterol can be used to introduce the biochemistry of life with and without oxygen (Brown and Galea, 2010). Cholesterol synthesis is an exceptionally oxygen-intensive process, which implies that its biosynthesis could have emerged only after the great oxygenation event (Rasmussen et al, 2008) through a rapid expansion of metabolic networks (Raymond and Segrè, 2006). Thus, this complex biochemical pathway allows students to explore the evolution of mono and di-oxygenase enzymes, how these enzymes carry out redox chemistry, learn how redox environments are maintained within cells, and understand the importance of metals in redox biology prior to the existence of cofactors such as NADH.

**Figure 2:**
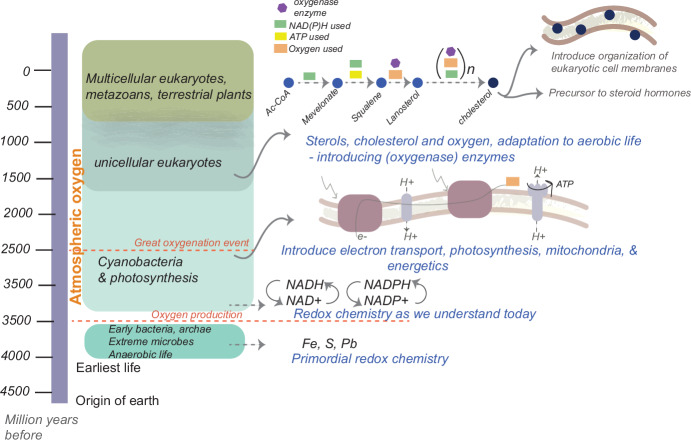
Biochemical evolution starting from atmospheric oxygen to redox cycles and the biosynthesis of cholesterol. A biochemistry teaching module built around cholesterol starts with the great oxygenation event, introduces principles of redox biochemistry before and after atmospheric oxygen, diverges to the evolution of electron transport and bioenergetics, and ends with the complex, oxygen-intensive synthesis of cholesterol and what it means for the origin and development of eukaryotes.

This module then transitions to the evolution of primordial energetics before an oxygen-rich atmosphere, the challenges, and opportunities that oxygen presented a cell with, and how ATP might have become a universal energy currency. This includes the relationships between oxygen use, specialized enzymes that make use of oxygen, the improbable success of the electron transport machinery, and cellular energetics as we understand it today. The module finally leaves students with an appreciation of why life and oxygen are so closely coupled along with a better understanding of the need for biochemical “cycles” (Fig. 2).

## Studying curiosities can end with the practical purpose

**Figure 3:**
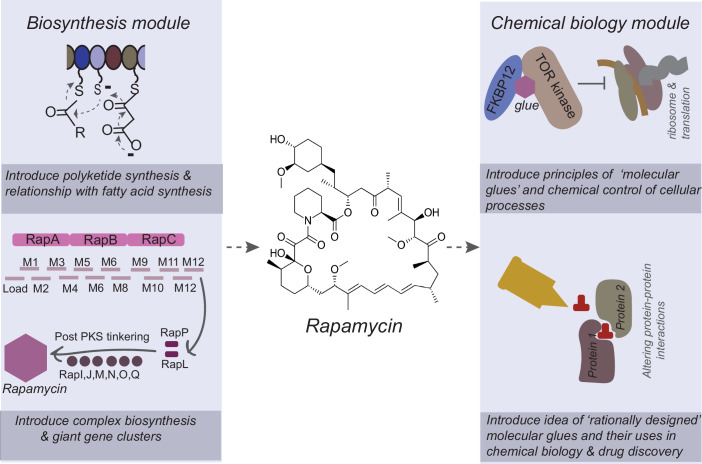
Rapamycin as an example to study the biochemistry behind complex biosynthesis and chemical biology. Using rapamycin as an example, students are introduced to the chemistry behind polyketide and non-ribosomal peptide synthesis, challenging biochemical reactions and complex biosynthetic pathways. Subsequently, rapamycin exemplifies a “molecular glue” and can be used to introduce different “chemical biology” approaches to target protein–protein interactions.

Another module used in the course is the story of Rapamycin. It is one of the most famous drugs, often used as an anti-aging agent, but here it becomes a starting point to introduce several concepts in chemical biology (Fig. 3). The module starts with a philosophical deviation, highlighting how important chance or serendipity is for scientific discovery (Koshland, 2007), while reiterating the role of dogged, curiosity-driven pursuit.

The story of rapamycin starts with its discovery by Surendranath Sehgal at Ayerst/Wyeth labs who analyzed soil samples from the Easter Islands, before introducing how such a ridiculously complex molecule is synthesized by bacteria, involving one of the largest gene clusters ever discovered (Park et al, 2010). It goes on to study the biochemical load this pathway imposes on the cell, and explore the idea of how the starting materials from amino and fatty acid precursors (CoA) might be synthesized through processes independent of the ribosome. Through this, we can now introduce the incredible chemical versatility of polyketide and non-ribosomal peptide synthesis (Payne et al, 2017) as engines of chemical diversity creation that are evolutionarily related to the fatty acid biosynthesis machinery. This introduces the world of “secondary metabolites”, and the modules of signaling and regulation these could enable in diverse niches.

Rapamycin also becomes an avenue to introduce genetic or biochemical approaches to identify a drug target (Heitman et al, 1991; Sabatini et al, 1994; Brown et al, 1994), while highlighting earlier chemical approaches to trap drugs with their natural ligands. An interesting curiosity is the idea of “molecular glues”: molecules that bring together two different proteins, which would otherwise never interact with one another, or have any role in regulating each other’s activity. How might have such molecules evolved, or been selected for?

Finally, the module transitions to practical ideas of molecular glues that are now being widely exploited in research (Neklesa et al, 2017), by engineering artificial compounds that bring proteins together and target them for degradation (Fig. 3). This module covers the whole space of “chemical ecology”, “chemical biology”, gene organization and biochemical evolution. All these modules only suggest the endless possibilities that biochemical curiosities present for teaching students.

## Conclusion

In a column titled *Biochemistry strikes back*, Sydney Brenner recalled his quip about Biochemistry: *“*Two things disappeared in 1990: one was communism, the other was biochemistry…Only one of these should be allowed to come back” (Brenner, 2000). Just in case you are unsure about the choice, the article continues with “[w]e do not have to resurrect biochemistry, and it will flourish because it provides the only experimental basis for causal understanding of biological mechanisms.” By highlighting biochemical curiosities, our course can hopefully pique the interest of students and inspire them to embrace biochemistry by asking why these mechanisms exist and how they may have evolved. We hope that this gives them a broader perspective to see the ‘forest beyond the leaves’ and to appreciate the complexity and wonders of the chemistry of life.

“By highlighting biochemical curiosities, our course can hopefully pique the interest of students and inspire them to embrace biochemistry by asking why these mechanisms exist and how they may have evolved.”

## Supplementary information

Peer Review File
