# Supplementary material for: Biochemical curiosities: Piquing students’ interest in biochemistry by exploring evolution’s pursuit of the improbable
Source: EMBO Rep. 2024 Sep 13;25(10):4100–4. doi: 10.1038/s44319-024-00259-7 (PMC11467204; doi:10.1038/s44319-024-00259-7)
Supplement: Supplementary file 1 — Peer Review File [file 44319_2024_259_MOESM1_ESM.pdf]

EMBO reports

Biochemical curiosities

Sunil Laxman

Corresponding author(s): Sunil Laxman (sunil@instem.res.in)

**Review Timeline:**	Submission Date:	7th Aug 24
	Accepted:	5th Sep 24

Editor: Holger Breithaupt

Transaction Report: This article was editorially reviewed.
